# Supplementary material for: Trainee psychotherapists’ emotion recognition accuracy during 1.5 years of psychotherapy education compared to a control group: no improvement after psychotherapy training
Source: PeerJ. 2023 Dec 11;11:e16235. doi: 10.7717/peerj.16235 (PMC10720477; doi:10.7717/peerj.16235)
Supplement: Supplemental Information 1 [file peerj-11-16235-s001.pdf]

Supplementary Material

Trainee psychotherapists' emotion recognition accuracy during 1.5 years of psychotherapy education compared to a control group: No improvement after psychotherapy training

Lillian Döllinger[1], M.Sc., Isabelle Letellier[2], Ph.D., Lennart Björn Högman[1], Ph.D., Petri Laukka[1], Ph.D., Håkan Fischer[1], Ph.D., & Stephan Hau[1], Ph.D.

Email address: lillian.dollinger@psychology.su.se

In the Supplementary Material, the reader finds information about the additional questionnaires that were used in the study, as well as descriptive statistics and group comparisons for the questionnaires (*Supplementary Table 1*). Spearman rank-order-correlations (Holm adjusted) were conducted for the ERA variables at pretest and follow-up and the questionnaires variables (see *Supplementary figure 1).* Further, we investigated the influence of age, gender and state affectivity on the ERA variables using linear regression analyses (see *Supplementary Table 2). Supplementary Table 3* reports the results of ERA group comparisons (trainee psychotherapists vs control group) regarding gender, and *Supplementary Table 4* reports results of group comparisons regarding ERA between the PDT and CBT students. *Supplementary Figure 2* shows the individual ERA change trajectories of the participants (observed data). Further, as sensitivity analyses for the mixed multilevel models with maximum likelihood estimation, we replicated the analyses without any missing data handling method purely based on the sample that performed both measurements (i.e., dropping the participants that dropped out from the analysis completely; see *Supplementary Table 5 & Supplementary Figure 3*). Exploratively, we added age as an additional predictor to the mixed multilevel models for *hypothesis 2*. Finally, we repeated the main analyses for *hypothesis 2* (mixed multilevel modeling) while differentiating between PDT and CBT trainees (PDT vs CBT vs control group), see *Supplementary Figure 4*.

Questionnaires

Participants of both groups filled in the *Experiences in Close Relationships-Revised* questionnaire (ECR-R; Fraley, Waller, & Brennan, 2000). The ECR-R assesses the adult attachment dimensions *avoidance* and *anxiety*. The participants judge 36 statements about how they generally experience romantic relationships using a 7-point Likert scale from 1 (*strongly disagree*) to 7 (*strongly agree*). We used a Swedish translation of the ECR-R.

The trainee psychotherapists were also administered a battery of other questionnaires. The *Therapeutic Identity Scale* (Sandell et al., 2004) was used to assess therapeutic attitudes. The questionnaire consists of 150 items in six sections. For this study, we did not look at items containing demographics or information about therapeutic orientation, supervision, own therapy and so on, but focused on two sections about therapeutic style. One section (33 items) is about curative factors and interventions in psychotherapy ("What Do You Think Contributes to Long-Term and Stable Therapeutic Change?") and included the factors *adjustment*, *insight* and *kindness*. The answering format is a 5-point Likert-scale ranging from 0 (*does not help at all*) to 4 (*helps a lot*). The other section is about individual therapeutic style factors ("What Are You Like as a Therapist?") and consists of the factors *neutrality*, *supportiveness* and *self-doubt*. Even this section contains a 5-point Likert-scale ranging from 0 (*do not agree at all*) to 4 (*agree very much*).

The *Self-Compassion Scale Short Format* (SCS-SF; Raes et al., 2011) is a 12-item questionnaire to assess self-compassion. A 5-point Likert-scale ranging from 1 (*almost never*) to 5 (*almost always*) the participants are to rate how they typically act towards themselves in difficult times. The questionnaire has 6 scales (*self-kindness*, *self-judgment*, *common humanity*, *isolation*, *mindfulness*, *over-identification*), but we used the average score of all items for this study.

The *Compassion Scale* (CS; Pommier, 2011) is a 24-item questionnaire with statements about how the person typically acts towards others and a 5-point Likert scale ranging from 1 (*almost never*) to 5 (*almost always*). The questionnaire has 6 scales (*kindness, indifference*, *common humanity*, *separation*, *mindfulness*, *disengagement*), but we used the average score of all items for this study.

Empathy was assessed using the Interpersonal Reactivity Index (IRI; Davis, 1983). This questionnaire consists of 28 statements about thoughts and feelings related to empathy and a 5-point Likert scale to indicate how much each statement describes the person well, ranging from 0 (*does not describe me well*) to 4 (*describes me very well*). The IRI scales are often divided into cognitive empathy (*perspective taking & fantasy*) and affective empathy (*empathic concern & personal distress)*.

Descriptive statistics, group comparisons and effect sizes for the questionnaire variables can be found in *Supplementary Table 1*.

Supplementary Table 1

Questionnaire variables: Descriptive statistics (means, standard deviations, 95% confidence intervals) and group comparisons (two-sided Wilcoxon-Mann-Whitney *U*-test)

**Measures**	**Trainee psychotherapists**	**Control group**	**Total**	***Statistic***	***Effect size***
	M (*SD*) [95% CI]	*M* (*SD*) [95% CI]	*M* (*SD*) [95% CI]	***t / U*** [95% CI]	***d / r*** [95% CI][b]
ECR-R: Attachment anxiety	2.77 (0.97) [2.49, 3.05]	2.91 (0.99) [2.72, 3.10]	2.87 (0.98) [2.70, 3.04]	*U* = 1715 *p* = .41 [-0.55, 0.22]	*r* = .07 [0.00, 0.24]
ECR-R: Attachment avoidance	2.39 (0.78) [2.17, 2.62]	2.67 (.0.96) [2.48, 2.85]	2.58 (0.91) [2.42, 2.74]	*U* = 1642 *p* = .17 [-0.61, 0.11]	*r* = .12 [0.01, 0.28]
Therapeutic Identity E1: Adjustment	1.80 (0.54) [1.62, 1.97]	*NA*			
Therapeutic Identity E1: Insight	1.94 (0.74) [1.70, 2.17]	*NA*			
Therapeutic Identity E1: Kindness	2.61 (0.67) [2.39, 2.83]	*NA*			
Therapeutic Identity E2: Neutrality	2.31 (0.31) [2.21, 2.41]	*NA*			
Therapeutic Identity E2: Supportiveness	2.24 (0.37) [2.12, 2.36]	*NA*			
Therapeutic Identity E2: Self-doubt	1.99 (0.42) [1.85, 2.13]	*NA*			
SCS-SF: Self-compassion	3.28 (0.34) [3.17, 3.38]	*NA*			
CS: Compassion	2.99 (0.17) [2.94, 3.05]	*NA*			
IRI: Cognitive empathy	2.16 (0.33) [2.06, 2.26]	*NA*			
IRI: Affective empathy	1.78 (0.28) [1.69, 1.86]	*NA*			

Note. [95% CI][b]: 95% Confidence Interval for effect size is based on 1000 bootstrap resamples of the mean difference (percentile interval). Common standardized effect size estimates: Wilcoxon-Mann-Whitney U-test: *r* = .10 (small), *r* = .30 (moderate), *r* = .50 (large). **p* < .05, ***p* < .01, ****p* < .001 (Holm adjusted).

Correlation matrix

Supplementary figure 1 displays the Spearman rank-order-correlations (Holm adjusted) among the ERA variables at pretest and follow-up and the questionnaires variables. The ERA variables mostly correlate with each other positively, apart from the MICRO follow-up that does not correlate with either ERAM score. The ERAM pre measurement shows a positive correlation with positive affectivity at follow up and negative correlations with anxious adult attachment, and the Therapeutic Identity scales adjustment and kindness. In the follow-up, the ERAM shows a positive correlation with negative affectivity at follow-up and affective empathy. The MICRO pre and follow-up scores do not correlate with any other variables than the ERA variables.

Variables possibly influencing ERA

We performed linear regression analyses to test for a possible influence of age, gender and state affectivity on ERA. Age and gender are known to influence ERA (see, e.g., Cortes et al., 2021; Thompson & Voyer, 2014) and even affective state has been found to lead to bias in emotion recognition (emotion congruent/incongruent mood bias), even if the results in this field are somewhat contradictory (see, e.g., Manierka et al., 2021; Schmid & Mast, 2010). Our results indicate that none of the variables significantly influenced ERA in this study (see *Supplementary Table 2*). Two-sample *t*-tests suggest no gender differences regarding ERA at either time point (see *Supplementary Table 3*). Further, we did not find ERA differences between the PDT and CBT students of the trainee psychotherapist group (see *Supplementary Table 4*).

Supplementary Table 2

Simple linear regression analyses of the influence of age, gender and state affectivity on the ERA variables

IV	DV	*b* [95% CI]	*SE b*	*β* [95% CI]	R^2 [95% CI]
Age	ERAM pre	.00 [-.00, .00]	.00	.11 [-.05, .27]	.01 [.00, .07]
Age	ERAM follow-up	-.00 [-.00, .00]	.00	-.06 [-.29, .18]	.00 [.00, .07]
Age	ERAM change-score	-.00 [-.00, .00]	.00	-.13 [-.37, .10]	.02 [.00, .12]
Age	MICRO pre	-.00 [-.00, .00]	.00	-.01 [-.17, .15]	.00 [.00, .02]
Age	MICRO follow-up	.00 [-.00, .00]	.00	.02 [-.22, .26]	.00 [.00, .05]
Age	MICRO change-score	.00 [-.00, .01]	.00	.07 [-.17, .30]	.00 [.00, .08]
Gender	ERAM pre	-.01 [-.04, .02]	.02		.00 [.00, .04]
Gender	ERAM follow-up	.01 [-.04, .06]	.02		.00 [.00, .06]
Gender	MICRO pre	-.04 [-.09, .01]	.03		.02 [.00, .07]
Gender	MICRO follow-up	.02 [-.06, .10]	.04		.00 [.00, .07]
PANAS pos pre	ERAM pre	.01 [-.01, .04]	.01	.08 [-.08, .23]	.01 [.00, .05]
PANAS pos follow-up	ERAM follow-up	.04 [-.01, .08]	.02	.25 [-.06, .56]	.06 [.00, .24]
PANAS neg pre	ERAM pre	.01 [-.03, .05]	.02	.04 [-.12, .20]	.00 [.00, .04]
PANAS neg follow-up	ERAM follow-up	.01	.04	.03	.00

		[-.07, .09]		[-.28, .34]	[.00, .07]
PANAS pos pre	MICRO pre	-.00	.02	-.01	.00
		[-.04, .04]		[-.17, .15]	[.00, .02]
PANAS pos follow-up	MICRO follow-up	-.01	.04	-.04	.00
		[-.09, .07]		[-.36, .28]	[.00, .09]
PANAS neg pre	MICRO pre	.01	.03	.02	.00
		[-.06, .08]		[-.14, .18]	[.00, .03]
PANAS neg follow-up	MICRO follow-up	.02	.07	.04	.00
		[-.12, .15]		[-.27, .35]	[.00, .09]

Note. *b* represents unstandardized regression weights; b*eta* indicates the standardized regression weights. *_p_ < .05, **_p_ < .01

Supplementary Table 3

Two-sided two-sample t-tests to explore gender (female/male) differences in ERA

	***t*(df)**	***p***	**95 % CI**
ERAM pre	t(152) = 0.59	.56	-0.02, 0.04
ERAM follow-up	t(70) = 0.35	.73	-0.05, 0.04
MICRO pre	t(152) = 1.55	.12	-0.01, 0.09
MICRO follow-up	t(70) = 0.43	.67	-0.10, 0.06

Note. *_p_ < .05, **_p_ < .01, ***_p_ < .001 (Holm adjusted).

Supplementary Table 4

Two-sided two-sample t-tests to explore differences in ERA between PDT and CBT students (only trainee psychotherapist group)

	***t*(df)**	***p***	**95 % CI**
ERAM pre	t(47) = 0.54	.59	-0.04, 0.06
ERAM follow-up	t(29) = -0.88	.39	-0.10, 0.04
MICRO pre	t(47) = 0.63	.54	-0.06, 0.12
MICRO follow-up	t(29) = -0.11	.91	-0.13, 0.12

Note. *_p_ < .05, **_p_ < .01, ***_p_ < .001 (Holm adjusted).

Individual ERA trajectories

In *Supplementary Figure 2*, the reader finds the ERA trajectories of the participants (observed data). Even though there was a clear trend for ERA improvement from pretest to follow-up, some participants did deteriorate.

Sensitivity analyses

Since there is no way to empirically verify whether data is missing at random or not, we chose to rerun the mixed multilevel modeling analyses even without a missing data handling method. For that, we completely excluded all participants that did not come back to the lab for the second measurement (n = 72). The results can be found in *Supplementary Table 5*. There were main effects of time for the ERAM as well as the MICRO, but no effects of group. More importantly, there were no *time* by *group* interactions for either outcome measure, suggesting that there were no between-group differences in slopes. The control group significantly improved in multimodal ERA from pretest (M = .41, SD = .09, 95% CI [.39 .45]) to follow-up (M = .45, SD = .10, 95% CI [.43 .47]), t(70) = -2.87, p = .03, 95% CI [-.07, -.00], whereas the trainee psychotherapists' multimodal ERA did not change from pretest (M = .44, SD = .08, 95% CI [.42 .47]) to follow-up up (M = .45, SD = .09, 95% CI [.42 .47]), t(70) = -0.29, p =.99, 95% CI [-.04, .04]. In micro expression ERA, the control group (pre: M = .46, SD = .14, 95% CI [.41 .50]; follow-up: M = .60, SD = .16, 95% CI [.57 .63]; t(70) = -6.02, p < .0001, 95% CI [-.21, -.08]), as well as the trainee psychotherapists (pre: M = .44, SD = .13, 95% CI [.40 .49]; follow-up: M = .54, SD = .16, 95% CI [.49 .58]; t(70) = -3.35, p = .01, 95% CI [-.17, -.02]) significantly improved. Even if the analyses with maximum likelihood estimation found significant differences in slopes and the present analyses did not, the post-hoc contrasts show the same pattern and the interpretation of the results is the same. The trainee psychotherapists did not improve in ERA more than the control group. The results of the analyses without the dropouts

underline the analyses using maximum likelihood estimation. *Supplementary Figure 3* provides a visual display of the results.

Supplementary Table 5

Fixed effects: Linear mixed-effects models

	Value	*SE*	*Df*	*t*-value	*p*-value	95% CI
ERAM (total)						
Intercept	0.42	0.01	70	29.84	.00***	0.39, 0.44
Time	0.04	0.01	70	2.87	.01**	0.01, 0.06
Group	0.03	0.02	70	1.32	.19	-0.01, 0.07
Time * Group	-0.03	0.02	70	-1.67	.10	-0.07, 0.01
MICRO						
Intercept	0.46	0.02	70	19.97	.00***	0.41, 0.50
Time	0.15	0.02	70	6.02	.00***	0.10, 0.19
Group	-0.01	0.03	70	-0.40	.69	-0.08, 0.05
Time * Group	-0.05	0.04	70	-1.43	.16	-0.13, 0.02

Note. Number of Observations: 144; Number of Groups: 72.
$^{*}p < .05$, $^{**}p < .01$, $^{***}p < .001$.

Explorative models regarding hypothesis 2

Even though age did not turn out to influence ERA at any time point (see *Supplementary Table 2*), the dropout was influenced by participants' age (see multiple logistic regression). For this reason, we added age as additional predictor in the mixed multilevel model analyses for hypothesis 2 as an explorative analysis (in addition to a random intercept, a fixed slope of time and group as predictor). In the ERAM model, age was not found to be a significant predictor of differences in ERA change between the groups (between-group difference in slope = 0.00, $SE = .00$, $t(151) = 0.12$, $p = .91$; 95% CI [-0.00, 0.00]) and the AIC was not indicating better model fit (AIC = -471.05). The same was true for the MICRO, (between-group difference in slope = -0.00, $SE = .00$, $t(151) = -0.25$, $p = .80$; 95% CI [-0.00, 0.00], AIC = -226.28. The models including also age were not statistically different from the full model including only the group * time prediction, $\chi^2(1) = 0.01$, $p = .90$ for the ERA and $\chi^2(1) = 0.07$, $p = .80$ for the MICRO.

In further exploratory analysis, we performed mixed multilevel model analyses in which we investigated differences in slopes between the PDT trainees, the CBT trainees and the control group (even if we had not found indication to believe that the PDT and CBT students significantly differed in their ERA in the *t*-tests; *Supplementary Table 4*). The alternative models included a random intercept, a fixed slope of time and group (PDT, CBT, CG) as predictor for multimodal and micro expression ERA. We found that the alternative models' fit was worse than the full models' (group being all trainee psychotherapists vs control group) with an AIC = -470.16 for the ERAM and an AIC = -224.71 for the MICRO. The full models and the alternative models did not differ from each other significantly, $\chi^2(2) = 1.13$, $p = .57$ for the ERAM and $\chi^2(2) = 0.50$, $p = .78$ for the MICRO. Because the sample sizes of the trainee psychotherapist subgroups would have been quite small after dividing them into PDT and CBT students (PDT = 34, CBT = 15) and because this was not our main research question, we did not present those analyses in the manuscript. However, the interested reader can find descriptive plots of the ERA changes below (*Supplementary Figure 4*).

References (supplementary)

Cortes, D.S., Tornberg, C., Bänziger, T. *et al.* (2021). Effects of aging on emotion recognition from dynamic multimodal expressions and vocalizations. *Sci Rep* 11, 2647. https://doi.org/10.1038/s41598-021-82135-1

Davis, M. H. (1983). Measuring individual differences in empathy: Evidence for a multidimensional approach. *Journal of Personality and Social Psychology, 44*(1), 113–126. https://doi.org/10.1037/0022-3514.44.1.113

Fraley, R. C., Waller, N. G., & Brennan, K. A. (2000). An item response theory analysis of self-report measures of adult attachment. *Journal of Personality and Social Psychology, 78*(2), 350–365. https://doi.org/10.1037/0022-3514.78.2.350

Manierka, M. S., Rezaei, R., Palacios, S., Haigh, S. M., & Hutsler, J. J. (2021). In the mood to be social: Affective state influences facial emotion recognition in healthy adults. *Emotion (Washington, D.C.), 21*(7), 1576–1581. https://doi.org/10.1037/emo0000999

Pommier, E. A. (2011). The compassion scale. *Dissertation Abstracts International Section A: Humanities and Social Sciences*, 72, 1174.

Raes, F., Pommier, E., Neff, K. D., & Van Gucht, D. (2011). Construction and factorial validation of ashort form of the Self-Compassion Scale. *Clinical Psychology & Psychotherapy*. 18, 250–255. https://doi.org/10.1002/cpp.702.

Sandell, R., Carlsson, J., Schubert, J., Broberg, J., Lazar, A., & Grant, J. (2004). Therapist attitudes and patient outcomes: I. Development and validation of the Therapeutic Attitudes Scales (TASC-2), *Psychotherapy Research*, 14:4, 469–484. http://dx.doi.org/10.1093/ptr/kph039.

Schmid, P. C., & Mast, M. S. (2010). Mood effects on emotion recognition. *Motivation and Emotion, 34*(3), 288–292. https://doi.org/10.1007/s11031-010-9170-0

Thompson, A. E., & Voyer, D. (2014). Sex differences in the ability to recognise non-verbal displays of emotion: a meta-analysis. *Cogn. Emot. 28,* 1164–1195. doi: 10.1080/02699931.2013.875889

Wei, T. & Simko, V. (2021). *R package 'corrplot': Visualization of a Correlation Matrix* (Version 0.92). Available from https://github.com/taiyun/corrplot.
