# Supplementary figures and images for: Trainee psychotherapists’ emotion recognition accuracy during 1.5 years of psychotherapy education compared to a control group: no improvement after psychotherapy training
Source: PeerJ. 2023 Dec 11;11:e16235. doi: 10.7717/peerj.16235 (PMC10720477; doi:10.7717/peerj.16235)

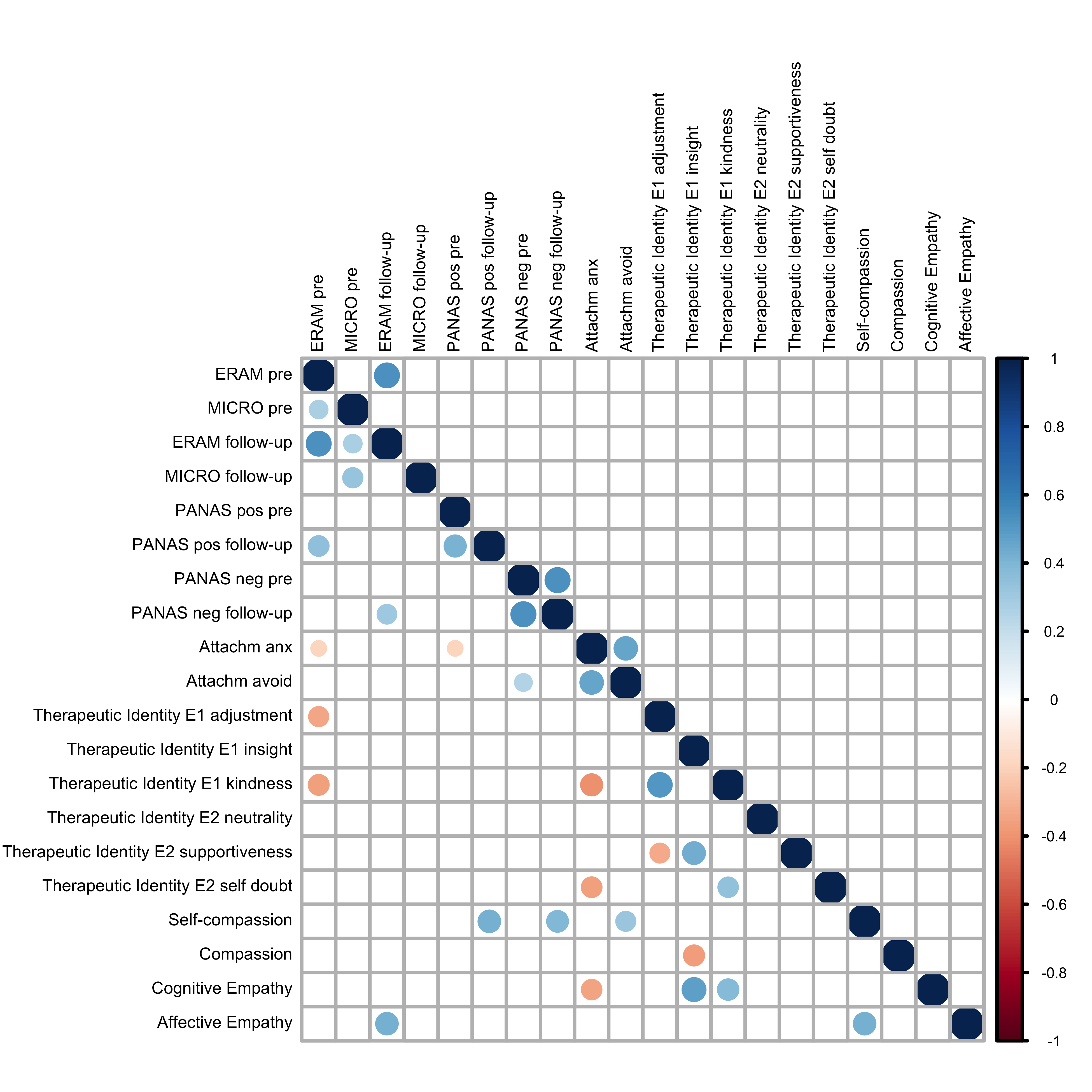

Supplement: Supplemental Information 2 — Note. Blue circles display positive correlations, red dots display negative correlations. The circle’s size and the intensity of the color (see scale on the right) provide information about the magnitude of the correlation. The significance level was set at 95%, blank fields indicate that there was no significant correlation (p > 0.05). We used the R package corrplot (Wei & Simko, 2021) for the visualization. [file peerj-11-16235-s002.png]

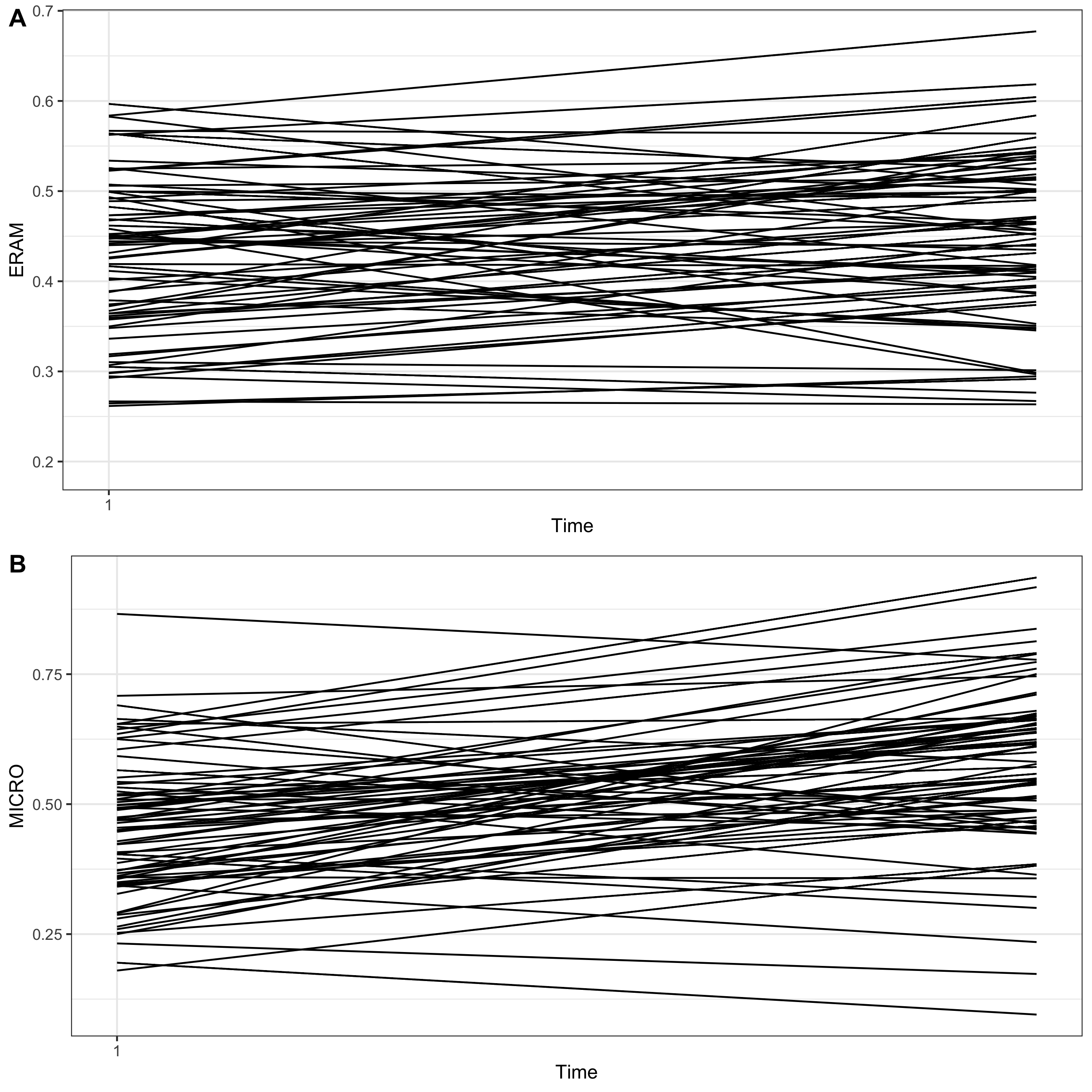

Supplement: Supplemental Information 3 — Note. N = 72, 0 = pretest, 1 = follow-up. [file peerj-11-16235-s003.png]

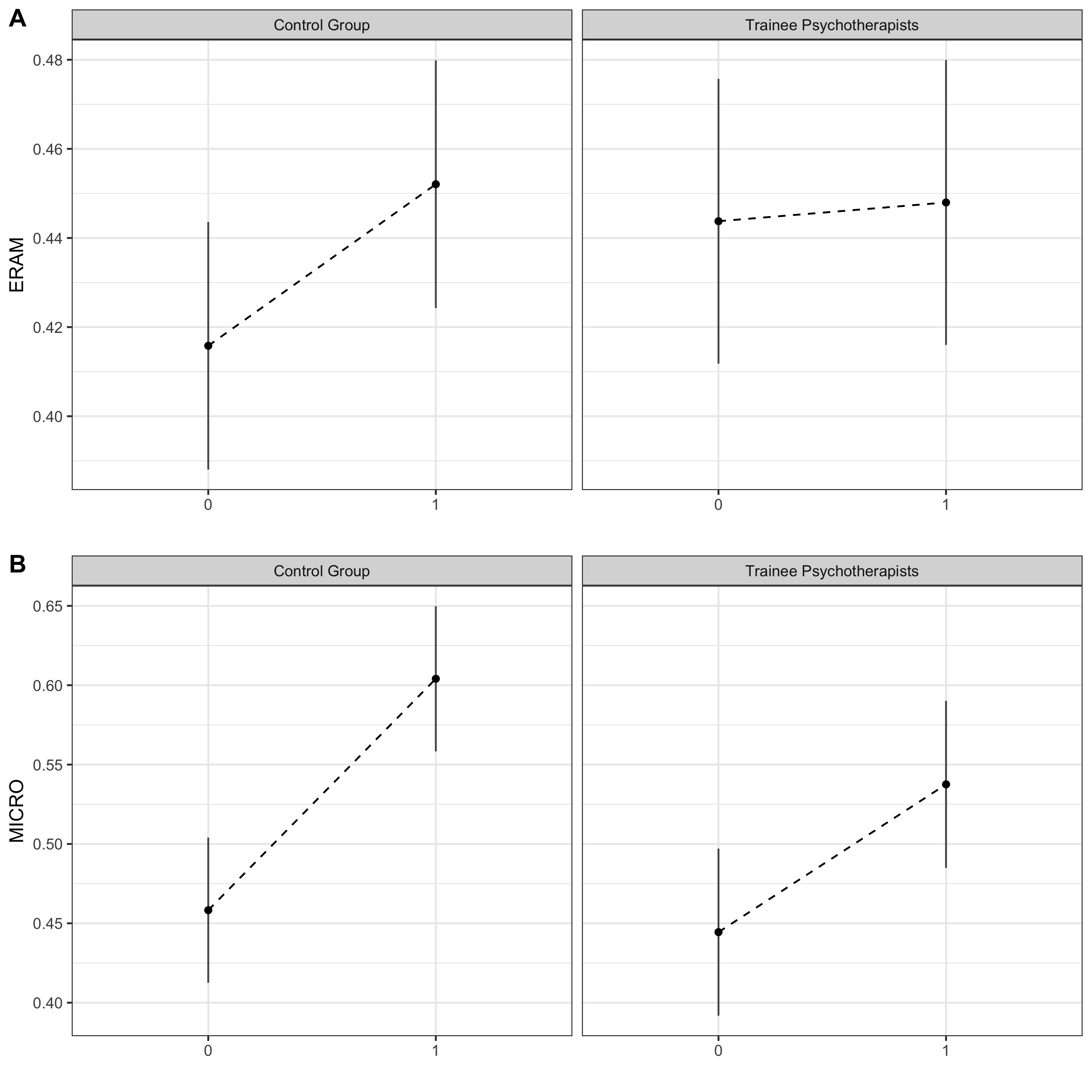

Supplement: Supplemental Information 4 — Note. N = 72 (after exclusion of dropouts). Error bars represent 95% Confidence Intervals. [file peerj-11-16235-s004.png]

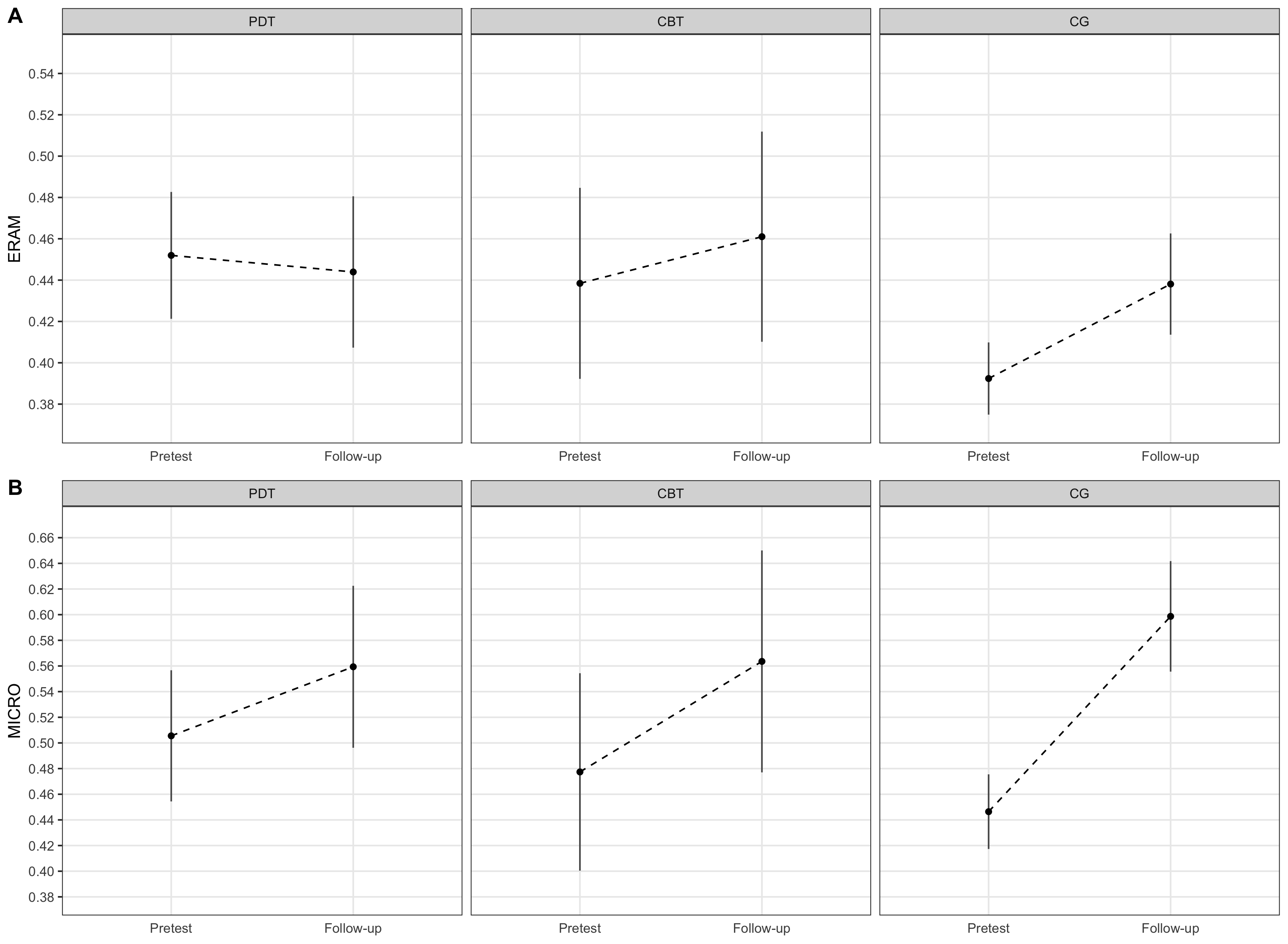

Supplement: Supplemental Information 5 — Note. Based on estimated marginal means. Error bars represent 95% Confidence Intervals. [file peerj-11-16235-s005.png]
